# Trimodality therapy versus perioperative chemotherapy in the management of locally advanced adenocarcinoma of the oesophagus and oesophagogastric junction (Neo-AEGIS): an open-label, randomised, phase 3 trial

**DOI:** 10.1016/S2468-1253(23)00243-1

**Published:** 2023-09-18

**Authors:** John V Reynolds, Shaun R Preston, Brian O'Neill, Maeve A Lowery, Lene Baeksgaard, Thomas Crosby, Moya Cunningham, Sinead Cuffe, Gareth O Griffiths, Imelda Parker, Signe Lenora Risumlund, Rajarshi Roy, Stephen Falk, George B Hanna, Frederick R Bartlett, Alberto Alvarez-Iglesias, Michael P Achiam, Magnus Nilsson, Guillaume Piessen, Narayanasamy Ravi, Dermot O'Toole, Ciaran Johnston, Raymond S McDermott, Richard C Turkington, Shajahan Wahed, Sharmila Sothi, Hugo Ford, Martin S Wadley, Derek Power, Neo-AEGIS Investigators and Trial Group, Somnath Mukherjee, Carys Morgan, Simon L Parsons, Neel Bhuva, Sorcha Campbell, Liam Grogan, Greg Leonard, Andrew R Bateman, Catherine Mitchell, Seamus O'Reilly, Eibhlin Mulroe, Olivia McLoughlin, Anna Shevlin, Aoife M Shannon, Jacinta Marron, Marc Nolan, Grace Burch, Michelle Costello, Daniel Griffiths, Kelly Cozens, Emma Foley, Claire L Donohoe, Catherine O'Farrell, Jennifer Moore, Jacintha O'Sullivan

**Affiliations:** a Cancer Trials Ireland, Dublin, Ireland; b St James's Hospital, Dublin, Ireland; c Royal Surrey County Hospital NHS Foundation Trust, Guildford, UK; d St Luke's Radiation Oncology Network, Dublin, Ireland; e Rigshopitalet, Copenhagen, Denmark; f Velindre University NHS Trust, Cardiff, UK; g Southampton Clinical Trials Unit, University of Southampton, Southampton, UK; h Hull University Teaching Hospitals NHS Trust, Hull, UK; i University Hospitals Bristol and Weston NHS Foundation Trust, Bristol, UK; j St Mary's Hospital, Imperial College, London, UK; k Portsmouth Hospitals University NHS Trust, Portsmouth, UK; l HRB Clinical Research Facility, NUI Galway, Galway, Ireland; m Division of Surgery, CLINTEC, Karolinska Institutet and Department of Upper Abdominal Diseases, Karolinska University Hospital, Stockholm, Sweden; n Claude Huriez University Hospital, Lille, France; o Belfast Health and Social Care Trust, Northern Ireland Cancer Centre, Belfast City Hospital, Belfast, UK; p Northern Oesophago-Gastric Unit, Royal Victoria Infirmary, Newcastle upon Tyne, UK; q University Hospitals Coventry and Warwickshire, Walsgrave, Coventry, UK; r Cambridge University Hospitals NHS Foundation Trust, Cambridge, UK; s Worcestershire Acute Hospitals NHS Trust, Worcestershire Oncology Centre, Worcestershire Royal Hospital, Worcester, UK; t Cork University Hospital, Wilton, Cork, Ireland

## Abstract

**Background:**

The optimum curative approach to adenocarcinoma of the oesophagus and oesophagogastric junction is unknown. We aimed to compare trimodality therapy (preoperative radiotherapy with carboplatin plus paclitaxel [CROSS regimen]) with optimum contemporaneous perioperative chemotherapy regimens (epirubicin plus cisplatin or oxaliplatin plus fluorouracil or capecitabine [a modified MAGIC regimen] before 2018 and fluorouracil, leucovorin, oxaliplatin, and docetaxel [FLOT] subsequently).

**Methods:**

Neo-AEGIS (CTRIAL-IE 10-14) was an open-label, randomised, phase 3 trial done at 24 centres in Europe. Patients aged 18 years or older with clinical tumour stage T2–3, nodal stage N0–3, and M0 adenocarcinoma of the oesophagus and oesophagogastric junction were randomly assigned to perioperative chemotherapy (three preoperative and three postoperative 3-week cycles of intravenous 50 mg/m^2^ epirubicin on day 1 plus intravenous 60 mg/m^2^ cisplatin or intravenous 130 mg/m^2^ oxaliplatin on day 1 plus continuous infusion of 200 mg/m^2^ fluorouracil daily or oral 625 mg/m^2^ capecitabine twice daily up to 2018, with four preoperative and four postoperative 2-week cycles of 2600 mg/m^2^ fluorouracil, 85 mg/m^2^ oxaliplatin, 200 mg/m^2^ leucovorin, and 50 mg/m^2^ docetaxel intravenously on day 1 as an option from 2018) or trimodality therapy (41·4 Gy in 23 fractions on days 1−5, 8−12, 15–19, 22–26, and 29–31 with intravenous area under the curve 2 mg/mL per min carboplatin plus intravenous 50 mg/m^2^ paclitaxel on days 1, 8, 15, 22, and 29). The primary endpoint was overall survival, assessed in all randomly assigned patients who received at least one dose of study drug, regardless of which study drug they received, by intention to treat. Secondary endpoints were disease-free survival, site of treatment failure, operative complications, toxicity, pathological response (complete [ypT0N0] and major [tumour regression grade 1 and 2]), margin-free resection (R0), and health-related quality of life. Toxicity and safety data were analysed in the safety population, defined as patients who took at least one dose of study drug, according to treatment actually received. The initial power calculation was based on superiority of trimodality therapy (n=366 patients); it was adjusted after FLOT became an option to a non-inferiority design with a margin of 5% for perioperative chemotherapy (n=540). This study is registered with ClinicalTrials.gov, NCT01726452.

**Findings:**

Between Jan 24, 2013, and Dec 23, 2020, 377 patients were randomly assigned, of whom 362 were included in the intention-to treat population (327 [90%] male and 360 [99%] White): 184 in the perioperative chemotherapy group and 178 in the trimodality therapy group. The trial closed prematurely in December, 2020, after the second interim futility analysis (143 deaths), on the basis of similar survival metrics and the impact of the COVID-19 pandemic. At a median follow-up of 38·8 months (IQR 16·3–55·1), median overall survival was 48·0 months (95% CI 33·6–64·8) in the perioperative chemotherapy group and 49·2 months (34·8–74·4) in the trimodality therapy group (3-year overall survival 55% [95% CI 47–62] *vs* 57% [49–64]; hazard ratio 1·03 [95% CI 0·77–1·38]; log-rank p=0·82). Median disease-free survival was 32·4 months (95% CI 22·8–64·8) in the perioperative chemotherapy group and 24·0 months (18·0–40·8) in the trimodality therapy group [hazard ratio 0·89 [95% CI 0·68–1·17]; log-rank p=0·41). The pattern of recurrence, locoregional or systemic, was not significantly different (odds ratio 1·35 [95% CI 0·63–2·91], p=0·44). Pathological complete response (odds ratio 0·33 [95% CI 0·14–0·81], p=0·012), major pathological response (0·21 [0·12–0·38], p<0·0001), and R0 rates (0·21 [0·08–0·53], p=0·0003) favoured trimodality therapy. The most common grade 3−4 adverse event was neutropenia (49 [27%] of 183 patients in the perioperative chemotherapy group *vs* 11 [6%] of 178 patients in the trimodality therapy group), followed by diarrhoea (20 [11%] *vs* none), and pulmonary embolism (ten [5%] *vs* nine [5%]). One (1%) patient in the perioperative chemotherapy group and three (2%) patients in the trimodality therapy group died from serious adverse events, two (one in each group) of which were possibly related to treatment. No differences were seen in operative mortality (five [3%] deaths in the perioperative chemotherapy group *vs* four [2%] in the trimodality therapy group), major morbidity, or in global health status at 1 and 3 years.

**Interpretation:**

Although underpowered and incomplete, Neo-AEGIS provides the largest comprehensive randomised dataset for patients with adenocarcinoma of the oesophagus and oesophagogastric junction treated with perioperative chemotherapy (predominantly the modified MAGIC regimen), and CROSS trimodality therapy, and reports similar 3-year survival and no major differences in operative and health-related quality of life outcomes. We suggest that these data support continued clinical equipoise.

**Funding:**

Health Research Board, Cancer Research UK, Irish Cancer Society, Oesophageal Cancer Fund, and French National Cancer Institute.

## Introduction

Adenocarcinoma of the oesophagus and oesophagogastric junction has increased in incidence in the western world over the past 40 years, fuelled by trends in gastrooesophageal reflux disease and obesity.^1^, ^2^ Curative approaches to locally advanced adenocarcinoma of the oesophagus and oesophagogastric junction are informed by randomised trials that established the superiority of both trimodality therapy, combining chemotherapy and radiotherapy before surgery, and preoperative or perioperative chemotherapy, compared with surgery alone.^3^, ^4^, ^5^, ^6^, ^7^ The pros and cons of each approach are frequently debated; however, no gold standard exists due to the scarcity of high-quality phase 3 trials. Accordingly, new data in this context would be valuable in the curative approach to a complex cancer.

Three trials have been central to this debate over the past decade. The CROSS randomised trial, published in 2012, compared trimodality therapy (radiotherapy with carboplatin plus paclitaxel) with surgery alone in patients with adenocarcinoma of the oesophagus and oesophagogastric junction or squamous cell carcinoma and reported a significant improvement in overall survival in the trimodality treatment group (median overall survival 48·6 months (95% CI 32·1–65·1) in the trimodality group and 24·0 months (14·2–33·7) in the surgery alone group (hazard ratio [HR] 0·68 [95% CI 0·53–0·88]; log-rank p=0·003); among the subgroup of patients with adenocarcinoma, median overall survival was 43·2 months (95% CI 24·9–61·4) in the trimodality group and 27·1 months (13·0–41·2) in the surgery alone group (3-year survival 54% [95% CI 47−64] *vs* 45% [38−54]; HR 0·73 [95% CI 0·55–0·98], log-rank p=0·038).^5^, ^8^ These finding provided the best reported outcome benchmark and consolidated international trends in patterns of care.^5^, ^8^, ^9^ For perioperative chemotherapy, the MAGIC trial, published in 2006, reported 5-year survival of 36·3% (95% CI 29·5–43·0) for patients with gastric adenocarcinoma or adenocarcinoma of the oesophagus and oesophagogastric junction treated with perioperative epirubicin, cisplatin, and fluorouracil (ECF) compared with 23·0% (95% CI 16·6–29·4) for those who underwent surgery alone.^5^ Modifications permitting oxaliplatin as a substitute for cisplatin and capecitabine as a substitute for fluorouracil (ie, the EOX regimen) subsequently became common practice, particularly in the UK. In 2019, the FLOT4-Arbeitsgemeinschaft Internistische Onkologie trial, also in patients with gastric adenocarcinoma or adenocarcinoma of the oesophagus and oesophagogastric junction, reported a significant survival benefit (median survival 50 months *vs* 35 months; HR 0·77 [95% CI 27·35–46·26) with perioperative fluorouracil, oxaliplatin, leucovorin, and docetaxel (FLOT) compared with ECF or EOX, which had a major impact on the choice of perioperative chemotherapy in these patient cohorts.^10^ However, the optimum approach to adenocarcinoma of the oesophagus and oesophagogastric junction—trimodality or perioperative chemotherapy—remains unknown.

Neo-AEGIS (Neoadjuvant trial in Adenocarcinoma of the Oesophagus and Oesophago-Gastric Junction International Study; also known as CTRIAL-IE 10-14) was designed after the publication of the CROSS trial, with a primary objective to directly compare the CROSS trimodality protocol (radiotherapy with carboplatin plus paclitaxel) with the modified MAGIC trial regimen (epirubicin plus cisplatin or oxaliplatin plus fluorouracil or capecitabine). A subtext was to address whether a regimen incorporating radiotherapy with a proven locoregional effect was required in an era with an increasing trend for radical en-bloc surgery and lymphadenectomy, and whether perioperative chemotherapy might have advantages through a reduction in rates of systemic failure. Moreover, although not evident in the CROSS trial, evidence from other trials and cohort studies that operative mortality and major respiratory morbidity might be increased in trimodality regimens represented an important question that required rigorous randomised analysis.^11^, ^12^, ^13^ The Neo-AEGIS trial design included unique elements, including a homogeneous population of patients with adenocarcinoma of the oesophagus and oesophagogastric junction, pre-treatment TNM staging with [^18^F]fluorodeoxyglucose ([^18^F]FDG)-CT-PET, radiotherapy quality assurance, and the strict reporting of operative complications according to the definitions of the Esophageal Complications Consensus Group (ECCG).^14^ The trial was amended after the publication of the FLOT trial to incorporate the FLOT regimen as an option, to reflect changes in the standard of care.

**Research in context**

**Evidence before the study**

We searched PubMed and the abstracts from major oncology congresses, including the European Society of Medical Oncology and the American Society of Clinical Oncology. For PubMed, we used full-text search terms for “oesophageal cancer”, “oesophagogastric cancer”, “adenocarcinoma of the oesophagus”, “adenocarcinoma of the oesophagogastric junction”, and “Siewert's cancer”. We searched for research articles published in English, initially from Jan 1, 1990, to Dec 31, 2012, before trial commencement, and regularly updated the search up to the June 30, 2023. International practice before Neo-AEGIS trial design in 2012 was based on published phase 3 randomised trials. The MAGIC trial, published in 2006, provided a standard bearer for perioperative chemotherapy (epirubicin plus cisplatin or oxaliplatin plus fluorouracil or capecitabine; the modified MAGIC regimen), and the CROSS trial, published in 2012, became the standard bearer for trimodality therapy (radiotherapy with carboplatin plus paclitaxel). Both trials showed a clear superiority of each approach compared with surgery alone. The only phase 3 trial of more than 100 patients (POET) comparing trimodality therapy and perioperative chemotherapy (n=119) did not complete and used a different regimen to that used in MAGIC and a lower radiation dose (30 Gy) than that used in CROSS (41·4 Gy). With the CROSS trial generating great interest, Neo-AEGIS set out to address this gap in knowledge, seeking out a gold standard, if one existed, and to provide data to inform international practice, which varies widely. Secondary endpoints were carefully chosen to inform clinician and patient choices if conclusive differences were not apparent in overall survival. The unique elements in trial design included the homogeneity of the study population, mandated staging with CT-PET, radiotherapy quality assurance, and reporting of operative complications using strictly defined criteria.

**Added value of the study**

This is the first randomised trial to compare the CROSS trimodality regimen with evidence-based perioperative chemotherapy (the modified MAGIC regimen, and from 2018 fluorouracil, leucovorin, oxaliplatin, and docetaxel [FLOT]). With strict inclusion criteria, and uniform staging, our data on 377 patients with locally advanced adenocarcinoma of the oesophagus and oesophagogastric junction inform the literature in several key dimensions despite the early trial termination. For the primary endpoint, similar survival estimates between both treatment groups at the first and second interim futility analyses, and with a median follow-up of longer than 3 years, should support clinical equipoise in decision making, particularly for a cancer for which most recurrences occur within 2 years. It was concluded by the data safety and monitoring board that more patients and longer follow-up would not changes survival metrics. Survival outcomes with trimodality therapy were similar to those in the CROSS trial; however, there was no evidence of superiority over perioperative chemotherapy. Trimodality therapy, as expected, was superior to perioperative chemotherapy in producing complete or major pathological responses and negative surgical margins. However, this did not translate to survival advantage, suggesting that radical en-bloc surgery was a key equalising factor. Perioperative chemotherapy did not reduce systemic failure rates compared with trimodality therapy, suggesting a modest effect on micrometastatic disease of the predominant modified MAGIC regimen, with no conclusions possible for FLOT. Trimodality therapy, despite concerns in this context from other randomised trials (POET and NeoRES) and large series in this context, did not increase operative mortality or major pulmonary morbidity. Both groups had similar health-related quality of life (HRQOL) at 1 and 3 years of follow-up; however, HRQOL was more adversely affected after trimodality therapy given before surgery than after perioperative chemotherapy.

**Implications of all the available evidence**

Neo-AEGIS set out to compare the trimodality regimen used in the CROSS trial with optimum perioperative chemotherapy. The similar survival metrics that was relevant to trial termination suggest that clinical equipoise between such approaches, specifically trimodality therapy and the modified MAGIC regimen or FLOT, is a reasonable conclusion. The limited FLOT treatment inclusion cannot be interpreted independently, hence the ESOPEC trial will be informative when published. Moreover, data relating to differences in toxicity, tolerance, and HRQOL, might help to inform clinicians and patients in treatment choices. In a dynamic evolving field, as we enter an era in which immunotherapy will be integrated with both these approaches, Neo-AEGIS provides a rich resource of randomised data. These data, as well as the range of secondary outcomes, will inform modern multidisciplinary team discussions and provide benchmark data for both treatment approaches.

## Methods

### Study design and participants

Neo-AEGIS was an investigator-initiated phase 3 trial done in 24 centres (appendix p 3) across Denmark, France, Ireland, Sweden, and the UK, sponsored by Cancer Trials Ireland.^15^ Only designated oesophageal centres were included for participation in the UK and Ireland, and regional centres in Europe. Patients aged 18 years or older with histological proof of adenocarcinoma of the oesophagus and oesophagogastric junction, and clinical tumour stage T2–3, nodal stage N0–3, and metastasis stage M0, were eligible.^16^ For junctional adenocarcinoma, patients with Siewert (AEG) types I, II, and III were eligible.^17^ All patients underwent initial staging with [^18^F]FDG-PET-CT imaging, and an endoscopic ultrasound was undertaken according to local standards. Only patients with primary tumours with a length of 8 cm or less, or with a combined length (tumour and nodes) for radiotherapy planning of 10 cm or less, were included. Patients were also required to have an Eastern Cooperative Oncology Group performance status of 0−2, a cardiac ejection fraction of greater than 50%, a forced expiratory volume in 1 s of 1·5 L or more on pulmonary function testing, absolute neutrophil count of more than 1·5 × 10^9^ cells per L, white blood cell count of more than 3 × 10^9^ cells per L, platelet count of more than 100 × 10^9^ per L, haemoglobin of more than 9 g/dL, glomerular filtration rate of more than 60 mL per min, serum bilirubin at the upper limit of normal (ULN) or below, aspartate aminotransferase of less than 2·5 times ULN, and alkaline phosphatase of less than 3 times the ULN. Patients who had undergone previous chemotherapy and radiotherapy were excluded. For the complete list of inclusion and exclusion criteria, see the appendix (p 7).

The trial was approved by the Health Products Regulatory Authority (Ireland), the Medicines and Healthcare products Regulatory Agency (UK), the Danish Medicines Agency, the French National Agency for the Safety of Medicines and Health Products, the Swedish Medical Products Agency, and by the relevant ethics committees. A trial steering committee and a data safety and monitoring board were appointed. Cancer Trials Ireland managed the trial, and the Health Research Board Clinical Research Facility at the National University of Ireland, Galway, Ireland conducted the data management and statistical activities in collaboration. The protocol is available online. All patients gave written informed consent.

### Randomisation and masking

Patients were randomly assigned (1:1) to receive perioperative chemotherapy or trimodality therapy. Randomisation sequences were generated using nQuery Advisor (version 6.01) using a permuted-block method to ensure a balance in sample size across the groups, with randomly permuted blocks of sizes two, four, six, and eight. There was no stratification, and the trial was open label.

### Procedures

Patients assigned to trimodality therapy received a preoperative radiation dose of 41·4 Gy given in 23 fractions of 1·8 Gy on days 1−5, 8−12, 15–19, 22–26, and 29–31, and 5 weekly cycles of paclitaxel (50 mg/m^2^) and carboplatin (area under the curve 2 mg/mL per min) intravenously on days 1, 8, 15, 22, and 29. Before a protocol amendment on May 1, 2018, patients assigned to perioperative chemotherapy received six (three preoperative and three postoperative) 3-week cycles of intravenous 50 mg/m^2^ epirubicin on day 1, plus either intravenous 60 mg/m^2^ cisplatin or intravenous 130 mg/m^2^ oxaliplatin on day 1, plus either continuous infusion of 200 mg/m^2^ fluorouracil daily for 21 days or oral 625 mg/m^2^ capecitabine twice daily for 21 days. According to a protocol amendment on May 1, 2018, patients in the perioperative chemotherapy group could alternatively receive FLOT: eight (four preoperative and four postoperative) 2-week cycles of 2600 mg/m^2^ fluorouracil, 85 mg/m^2^ oxaliplatin, 200 mg/m^2^ leucovorin, and 50 mg/m^2^ docetaxel intravenously on day 1.^5^, ^6^, ^7^ The choice of MAGIC or FLOT regimen was at the treating oncologist's discretion, and dose reductions, dose modifications, and supportive therapy were permitted according to the protocol. Radiotherapy quality assurance involved all centres completing pre-accrual outlining benchmark cases, a satisfactory plan for a pre-outlined patient, and a process document detailing technical elements of all trial patient processes for that centre.^15^ The first case from each centre was reviewed, and a random allocation of 10% of all outlines and plans was submitted. Radiotherapy quality assurance was overseen by two radiotherapy principal investigators: BO'N (Ireland and Europe) and TC (UK).

Restaging by means of endoscopy and [^18^F]FDG-PET-CT or CT was performed 4−8 weeks after completion of neoadjuvant therapy. Endoscopic response before surgery was defined as complete, partial (>50% tumour reduction), and none (<50% tumour reduction). Surgery was scheduled between 3 weeks and 10 weeks after the last treatment. Disease recurrence was monitored via CT or CT/PET imaging and endoscopy at 1, 2, and 3 years from the date of randomisation. Where recurrence was suspected, [^18^F]FDG-PET-CT was performed, and where recurrence was equivocal, cytological or histopathological confirmation was sought. Site of initial recurrence included locoregional at the site of anastomosis or regional nodes, or systemic, or both.

### Outcomes

The primary endpoint was overall survival calculated from the date of randomisation to the date of death from any cause, and those alive at time of termination were censored at time of the last assessment. Secondary endpoints were disease-free survival, site of treatment failure, operative complications in the first 90 days after surgery (according to ECCG-defined complication definitions^14^ and the Clavien−Dindo severity classification^18^), toxicity (according to National Cancer Institute Common Terminology Criteria for Adverse Events, version 4.03), pathological complete response (ypT0N0); major pathological response (tumour regression grade 1 and 2, denoting no [1] or minimal [2] residual cancer cells),^19^ margin-free resection (R0 classification), and health-related quality of life (HRQOL).^20^ HRQOL was measured using the European Organisation for Research and Treatment of Cancer (EORTC) core questionnaire QLQ-C30 (version 3.0) consisting of global health and five functional scales, and the QLQ-OES 18 module of four scales and six single items. These questionnaires were completed by patients at diagnosis, preoperatively, and at 3 months, 6 months, 1  year, 2 years, and 3 years of follow-up.^21^ A mean difference of 10 in global and functional scales is deemed to be clinically significant.^21^ Time to treatment failure was included in the original protocol; however, it is not reported independently because regimen completion data, disease-free survival, and overall survival provide the key relevant time to treatment failure metrics.

### Statistical analysis

The initial design in 2013 was based on a two-sided α level of 0·05 and an estimated 80% power to detect a 3-year increase in overall survival of 15% (58% with trimodality therapy *vs* 43% with perioperative chemotherapy), with trimodality being predicted superior to the comparator perioperative chemotherapy, and targeting 366 patients. This design was modified in 2014 to a 10% increase (53% with trimodality therapy *vs* 43% with perioperative chemotherapy), requiring 594 patients to achieve 80% power at a 0·05 significance level, corresponding to a hazard ratio (HR) of 1·33 (appendix p 5). An amendment on May 1, 2018, permitted the use of the FLOT regimen in the perioperative chemotherapy group and resulted in a change to a non-inferiority design. The first futility analysis on Dec 4, 2018, after 76 deaths, revealed an anticipated 3-year survival of 57% for perioperative chemotherapy and 52% for trimodality therapy. This resulted in a revised power calculation based on an expected 3-year survival of 57% for perioperative chemotherapy and 53% for trimodality therapy, with a non-inferiority margin for perioperative chemotherapy of 5%, corresponding to a non-inferiority HR of 1·16 with perioperative chemotherapy as the numerator, and an α level of 5%. The revised enrolment target was 540 patients. Neo-AEGIS closed in December, 2020, following the second futility analysis (143 deaths; 50% of expected deaths), with 377 patients randomly assigned (70% of the target). Although futility and non-inferiority were not established, the data safety and monitoring board determined that the similar survival rates from both interim analyses were highly unlikely to change with more patients or longer-term follow-up. The impact of the COVID-19 pandemic was a further factor in this.

All efficacy analyses were done in the intention-to-treat population, defined as all randomly assigned patients who received at least one dose of study drug. The intention-to-treat analyses were performed using treatment allocated regardless of dosing errors. Toxicity and safety data were analysed in the safety population, defined as patients who took at least one dose of study drug, according to treatment actually received.

Overall survival was calculated from randomisation to death from any cause. Disease-free survival was calculated from randomisation to the first event (ie, local recurrence or progression, distant recurrence, or death from any cause). We compared survival times between the two groups using the log-rank test and a Cox regression analysis to obtain a HR with a 95% CI. A multivariate analysis was used to calculate HRs and 95% CIs adjusted for baseline age, sex, smoking status, and tumour staging. The χ^2^ test (or Fisher's exact test for small counts) was used for categorical data, and appropriate parametric *t* tests or non-parametric Mann-Whitney tests were used otherwise. All tests are two sided unless stated otherwise. Interim futility analyses applied the rule of Freidlin and colleagues with a linear 20% inefficacy boundary and were planned a priori for 25% and 50% predicted deaths. ^22^ The primary endpoint was checked for non-inferiority; all other p values reported are for superiority. Analysis performed using SAS (version 9.4) and R (version 4.1.2).

This study is registered with ClinicalTrials.gov, NCT01726452.

### Role of the funding source

The funders of the study had no role in the study design, data collection, data analysis, data interpretation, or writing of the report.

## Results

**Figure 1:**
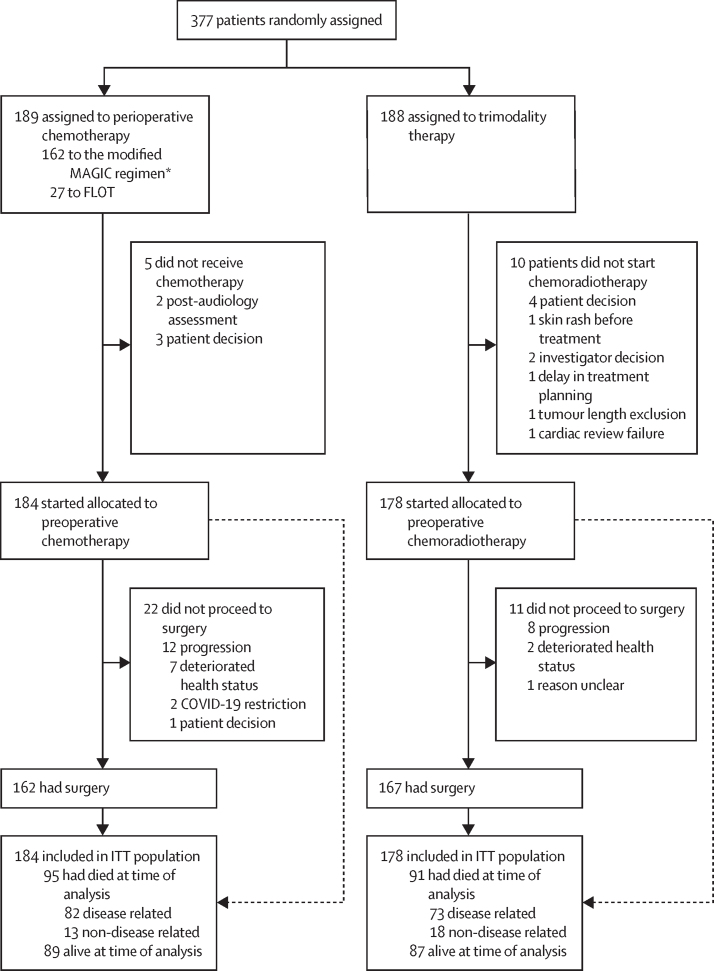
Trial profile FLOT=fluorouracil, leucovorin, oxaliplatin, and docetaxel. ITT=intention to treat. *Epirubicin plus cisplatin or oxaliplatin plus fluorouracil or capecitabine.

Between Jan 24, 2013, and Dec 23, 2020, 189 patients were randomly assigned to the perioperative chemotherapy group and 188 to the trimodality therapy group (figure 1). 184 patients in the perioperative chemotherapy group and 178 in the trimodality therapy group started treatment and were included in the intention-to-treat analyses (327 [90%] male and 360 [99%] White). 27 (15%) patients in the perioperative chemotherapy group received the FLOT regimen. Baseline characteristics are shown in table 1. 249 (69%) patients had oesophageal adenocarcinoma or AEG type I, 305 (84%) had clinical stage T3, and 211 (58%) had clinical predicted node-positive (cN+) disease. In the perioperative chemotherapy group, 166 (90%) of 184 patients completed all preoperative cycles, 108 (59%) completed at least one postoperative cycle, and 75 (41%) received the complete regimen (ie, all six [modified MAGIC regimen] or eight [FLOT] cycles); one patient had surgery but not chemotherapy. In the trimodality therapy group, 154 (87%) of 178 patients completed the full treatment regimen and 177 (99%) completed the entire radiotherapy regimen. Patients in the perioperative chemotherapy group were significantly more likely to have a dose reduction than those in the trimodality therapy group (75 [41%] *vs* 16 [9%] patients; odds ratio [OR] 6·94 [95% CI 3·84–12·56], p<0·0001). Fewer patients in the trimodality therapy group withdrew from treatment due to toxicity than those in the perioperative chemotherapy group, although this difference did not reach significance (25 [14%] *vs* 14 [8%]; OR 0·54 [95% CI 0·27–1·08], p=0·077). 165 (46%) of 362 patients had at least one serious adverse event (91 [50%] in the perioperative chemotherapy group and 74 [42%] in the trimodality therapy group (table 2). Grade 3 or 4 neutropenia was observed in 49 (27%) of 183 patients in the perioperative chemotherapy group and 11 (6%) of 178 patients in the trimodality therapy group (p<0·0001). One (1%) patient in the perioperative chemotherapy group and three (2%) patients in the trimodality therapy group died as a results of serious adverse events, two of which were possibly related to treatment: a cerebrovascular accident in the perioperative chemotherapy group and pulmonary embolism in the trimodality therapy group.

**Table 1:** Baseline characteristics

		**Perioperative chemotherapy group (n=184)**	**Trimodality therapy group (n=178)**
Age, years		63·8 (8·8)	63·8 (7·9)
Sex			
	Male	169 (92%)	158 (89%)
	Female	15 (8%)	20 (11%)
Race			
	White	183 (99%)	177 (99%)
	Other	1 (1%)	1 (1%)
BMI, kg/m^2^		27·5 (3·9)	27·8 (4·5)
Diabetes		15 (8%)	23 (13%)
Hypertension		68 (37%)	60 (34%)
Current smoker		23 (13%)	20 (11%)
ECOG performance status			
	0	155 (84%)	148 (83%)
	1	27 (15%)	28 (16%)
	2	2 (1%)	2 (1%)
Tumour location			
	Lower oesophagus or AEG type I	123 (67%)	126 (71%)
	AEG type II	46 (25%)	38 (21%)
	AEG type III	15 (8%)	14 (8%)
Clinical tumour stage			
	T2	29 (16%)	28 (16%)
	T3	155 (84%)	150 (84%)
Clinical nodal stage			
	N0	73 (40%)	78 (44%)
	N1	83 (45%)	73 (41%)
	N2	23 (13%)	27 (15%)
	N3	5 (3%)	0
Surgery type			
	En-bloc two-stage transthoracic resection	115/162 (80%)	130/167 (78%)
	Minimally invasive en-bloc	30/162 (19%)	17/167 (10%)
	En-bloc three-stage transthoracic resection	7/162 (4%)	4/167 (2%)
	Extended total gastrectomy and mediastinal anastomosis	6/162 (4%)	6/167 (4%)
	Extended total gastrectomy and thoracic anastomosis	2/162 (1%)	3/167 (2%)
	Trans-hiatal oesophagectomy	2/162 (1%)	7/167 (4%)

Data are mean (SD), n (%), or n/N (%). Percentages might not sum to 100 as a result of rounding. AEG=adenocarcinoma of the oesophagogastric junction. ECOG=Eastern Cooperative Oncology Group.

**Table 2:** Potentially chemotherapy or radiotherapy-associated adverse events (whether related or not) assessed in the safety population by treatment group

	Perioperative chemotherapy group (n=183*)			**Trimodality therapy group (n=178)**			**p value**
	Grade 1 or 2	Grade 3 or 4	Grade 5	Grade 1 or 2	Grade 3 or 4	Grade 5	
At least one serious adverse event	14 (8%)	71 (39%)	6 (3%)	9 (5%)	57 (32%)	8 (4%)	..
Diarrhoea	79 (43%)	20 (11%)	0	46 (26%)	0	0	<0·0001
Vomiting	52 (28%)	14 (8%)	0	34 (19%)	5 (3%)	0	0·0007
Nausea	105 (57%)	10 (5%)	0	96 (54%)	8 (4%)	0	0·68
Fatigue	114 (62%)	5 (3%)	0	101 (57%)	2 (1%)	0	0·25
Constipation	71 (39%)	2 (1%)	0	78 (44%)	2 (1%)	0	0·60
Odynophagia	5 (3%)	0	0	37 (21%)	5 (3%)	0	<0·0001
Neutropenia	56 (31%)	49 (27%)	0	19 (11%)	11 (6%)	0	<0·0001
Anaemia	22 (12%)	4 (2%)	0	8 (4%)	2 (1%)	0	0·018
Neutropenic sepsis	0	4 (2%)	0	0	1 (1%)	0	0·37
Peripheral neuropathy	65 (35%)	4 (2%)	0	13 (7%)	0	0	<0·0001
Alopecia	46 (25%)	1 (0·5%)	0	14 (8%)	0	0	<0·0001
Infections	33 (18%)	15 (8%)	0	37 (21%)	16 (9%)	0	0·85
Pulmonary embolism	2 (1%)	10 (5%)	0	1 (1%)	9 (5%)	1 (1%)	1

Data are n (%). Serious adverse events are reported for all patients in the safety population and include postoperative mortality events.

* One patient in the intention-to-treat population had surgery but not chemotherapy and was therefore excluded from the safety population.

**Figure 2:**
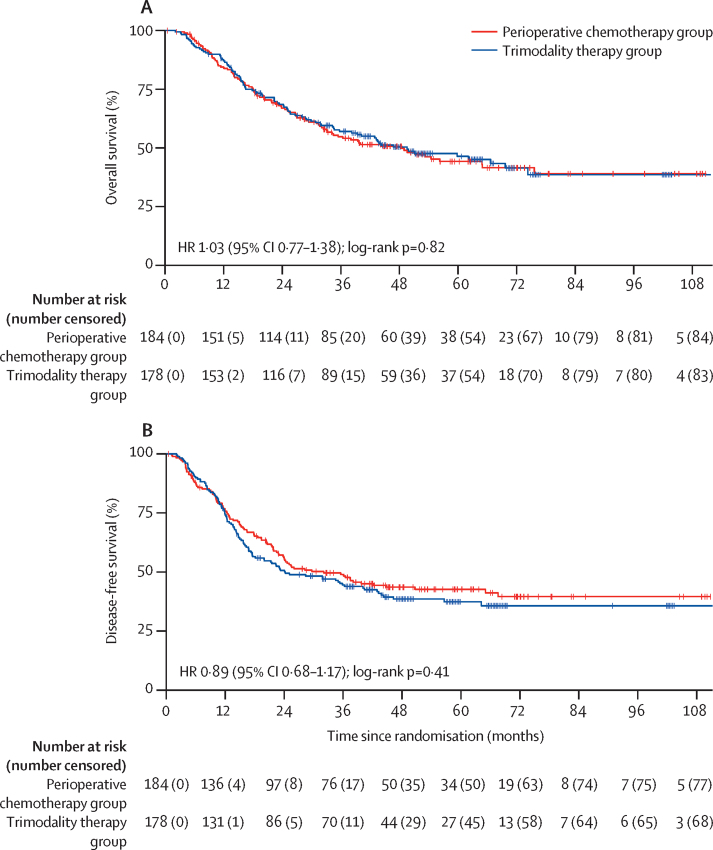
Kaplan-Meier estimates of overall survival and disease-free survival in the intention-to-treat population (A) Overall survival. (B) Disease-free survival. HR=hazard ratio.

Minimum follow-up was 18 months after the last recruited patient. The final data analysis was done on March 22, 2023. Median follow-up was 38·8 months (IQR 16·3–55·1). In the intention-to-treat population, in the perioperative chemotherapy group, 95 (52%) of 184 patients died: 82 (45%) from recurrent or progressive cancer, seven (4%) from non-cancer-related deaths, five (3%) from postoperative mortality, and one (1%) from a serious adverse event. In the trimodality therapy group, 91 (51%) of 178 patients died: 73 (41%) from cancer recurrence or progression, 11 (6%) from non-cancer-related deaths, four (2%) from postoperative complications, and three (2%) from serious adverse events. Median overall survival was 48·0 months (95% CI 33·6–64·8) in the perioperative chemotherapy group versus 49·2 months (34·8–74·4) in the trimodality therapy group (figure 2; HR 1·03 [95% CI 0·77–1·38], p=0·82). 1-year overall survival was 84% (95% CI 78–89) in the perioperative chemotherapy group versus 87% (81–91) in the trimodality therapy group, 2-year overall survival was 67% (59–73) versus 69% (61–75), and 3-year overall survival 55% (47–62) versus 57% (49–64).

Median disease-free survival was 32·4 months (95% CI 22·8–64·8) in the perioperative chemotherapy group and 24·0 months (18·0–40·8) in the trimodality therapy group (HR 0·89 [95% CI 0·68–1·17], p=0·41).

In the perioperative chemotherapy group, of 72 (39%) of 184 patients with progression or recurrence, 65 died, with the site of first failure being locoregional in 18 (10%) patients, systemic in 39 (21%) patients, or combined in 15 (8%) patients (table 3). In the trimodality therapy group, among 81 (46%) of 178 patients with progression or recurrence, 65 died, with the site of first failure being locoregional in 16 (9%) patients, systemic in 41 (23%) patients, or combined in 24 (13%) patients. There was no significant difference in locoregional alone versus systemic alone or combined recurrence between treatment groups (18 [25%] of 65 had locoregional recurrence in the perioperative chemotherapy group compared with 16 [20%] of 65 in the trimodality therapy group; OR 1·35 [95% CI 0·63–2·91]; p=0·44).

**Table 3:** Pathological response, tumour regression grade, resection margin status, response to therapy, and site of treatment failure

		**Perioperative chemotherapy group (n=162)**	**Trimodality therapy group (n=167)**	**p value**
Tumour pathology		..	..	0·020
	ypT0	7 (4%)	23 (14%)	..
	ypT1a	6 (4%)	8 (5%)	..
	ypT1b	19 (12%)	26 (16%)	..
	ypT2	24 (15%)	22 (13%)	..
	ypT3	97 (60%)	84 (50%)	..
	ypT4	9 (6%)	4 (2%)	..
Nodal pathology		..	..	0·0035
	ypN0	71 (44%)	100 (60%)	..
	ypN1	50 (31%)	35 (21%)	..
	ypN2	16 (10%)	21 (13%)	..
	ypN3	25 (15%)	11 (7%)	..
Tumour regression grade		..	..	<0·0001
	1	8 (5%)	23 (14%)	..
	2	11 (7%)	41 (25%)	..
	3	38 (23%)	53 (32%)	..
	4	65 (40%)	39 (23%)	..
	5	35 (22%)	7 (4%)	..
	Not evaluable	5 (3%)	4 (2%)	..
Pathological complete response		7 (4%)	20 (12%)	0·012
Circumferential margin R0		119/145 (82%)	131/137 (96%)	0·0003
Number of nodes analysed		27 (22–37)	22 (16–31)	0·0002
Number of nodes involved		1 (0–3)	0 (0–2)	0·0025
Response to therapy by endoscopy		..	..	0·020
	Complete response	23/130 (18%)	28/138 (20%)	..
	Partial response	62/130 (48%)	83/138 (60%)	..
	No response	45/130 (35%)	27/138 (20%)	..
Site of treatment failure (multiple sites possible per patient)				
	Systemic	49/184 (27%)	58/178 (33%)	..
	Liver	11/184 (6%)	22/178 (12%)	0·035
	Lung	13/184 (7%)	24/178 (13%)	0·044
	Bone	12/184 (7%)	17/178 (10%)	..
	Multiple sites	22/184 (12%)	26/178 (15%)	..
	Nodal non-regional	14/184 (8%)	20/178 (11%)	..
	Locoregional	27/184 (15%)	34/178 (19%)	..
	Anastomosis and oesophageal	17/184 (9%)	21/178 (12%)	..
	Stomach	6/184 (3%)	2/178 (1%)	..
	Regional nodes	15/184 (8%)	17/178 (10%)	..
	Missing	1/184 (1%)	1/178 (1%)	..

Data are n (%), n/N (%), or median (IQR). Percentages might not sum to 100 as a result of rounding.

A greater proportion of patients in the trimodality group had a pathological complete response compared with the perioperative chemotherapy group (OR 0·33 [95% CI 0·14–0·81], p=0·012; table 3). Similarly, major pathological responses, comprising tumour regression grade 1 and 2 combined, were seen in more patients in the trimodality therapy group than in the perioperative chemotherapy group (OR 0·21 [95% CI 0·12–0·38], p<0·0001; table 3). A combined poor (tumour regression grade 4) or absent (tumour regression grade 5) response was seen in a greater proportion of patients in the perioperative chemotherapy group than in the trimodality therapy group (OR 4·46 [95% CI 2·79–7·15], p<0·0001**;** table 3). Negative margins (R0) were observed in a greater proportion of patients in the trimodality therapy group than in the perioperative chemotherapy group (OR 0·21 [95% CI 0·08–0·53], p=0·0003; table 3). A higher proportion of tumours were ypN0 in the trimodality therapy group than in the perioperative chemotherapy group (OR 0·52 [95% CI 0·34–0·81], p=0·0035; table 3). Response to therapy as determined by endoscopy was significantly different between treatment groups (χ^2^ p=0·020), with a higher proportion of partial responses in the trimodality therapy group than in the perioperative chemotherapy group and a lower proportion of no response cases (table 3). Complete endoscopic responses were similar in both groups (table 3).

162 (88%) of 184 patients in the perioperative chemotherapy group and 167 (94%) of 178 patients in the trimodality therapy group underwent surgery, the majority via an en-bloc open or minimally invasive transthoracic resection (table 1). At 90 days, five (3%) of 162 patients in the perioperative chemotherapy group and four (2%) of 167 patients in the trimodality therapy group had died. Complications including pneumonia, respiratory failure, atrial fibrillation, and anastomotic leaks were similar in the perioperative chemotherapy and trimodality therapy groups (table 4). Severe complications according to the Clavien–Dindo classification, including grade IIIb (requiring invasive operative management), IVa (single organ failure), and IVb (multi-organ failure), occurred in 17 (11%) patients in the perioperative chemotherapy group and 21 (13%) patients in the trimodality therapy group (OR 0·82 [95% CI 0·41–1·61], p=0·56).

**Table 4:** Postoperative complications 90 days after surgery according to Esophageal Complications Consensus Group definitions^14^ and the Clavien–Dindo severity classification^18^

	**Perioperative chemotherapy group (n=162)**	**Trimodality group (n=167)**
**Gastrointestinal**		
Oesophagogastric leak from anastomosis, staple line or localised conduit necrosis	18 (11%)	19 (11%)
Extensive conduit necrosis	1 (1%)	0
Ileus	0	2 (1%)
Small bowel obstruction	0	2 (1%)
Feeding J-tube complication	2 (1%)	2 (1%)
Pyloromyotomy or pyloroplasty complication	1 (1%)	0
*Clostridioides difficile* infection	1 (1%)	2 (1%)
Pancreatitis	0	1 (1%)
Gastrointestinal bleeding requiring intervention or transfusion	2 (1%)	2 (1%)
Liver dysfunction	4 (2%)	2 (1%)
Delayed gastric emptying requiring intervention or delaying discharge or requiring maintenance of nasogastric drainage >7 days after operation	5 (3%)	2 (1%)
**Pulmonary**		
Pneumonia	32 (20%)	26 (16%)
Pleural effusion requiring additional drainage procedure	18 (11%)	25 (15%)
Pneumothorax requiring intervention	9 (6%)	7 (4%)
Atelectasis mucous plugging requiring bronchoscopy	3 (2%)	1 (1%)
Respiratory failure requiring reintubation	13 (8%)	13 (8%)
Acute respiratory distress syndrome	1 (1%)	7 (4%)
Acute aspiration	4 (2%)	3 (2%)
Tracheobronchial injury	1 (1%)	2 (1%)
Chest tube drainage for >10 days after operation	6	9
**Cardiac**		
Myocardial infarction	1 (1%)	0
Atrial dysrhythmia requiring intervention	21 (13%)	26 (16%)
Ventricular dysrhythmia requiring intervention	1 (1%)	1 (1%)
Congestive heart failure requiring intervention	2 (1%)	0
Pericarditis requiring intervention	1 (1%)	0
**Thromboembolic**		
Deep vein thrombosis	0	1 (1%)
Pulmonary embolus	7 (4%)	4 (2%)
Stroke	1 (1%)	3 (2%)
**Urological**		
Acute renal failure requiring dialysis	2 (1%)	1 (1%)
Urinary tract infection	1 (1%)	5 (3%)
Urinary retention requiring reinsertion of urinary catheter	5 (3%)	0
**Infection**		
Wound Infection requiring opening wound or antibiotics	9 (6%)	7 (4%)
Central intravenous line infection requiring removal or antibiotics	1 (1%)	0
Intrathoracic or intra-abdominal abscess	3 (2%)	5 (3%)
Generalised sepsis according to CDC definition	10 (6%)	8 (5%)
Other infections requiring antibiotics	23 (14%)	23 (14%)
**Neurological or psychiatric complications**		
Recurrent laryngeal nerve injury	4 (2%)	2 (1%)
Acute delirium	9 (6%)	7 (4%)
**Wound or diaphragm complications**		
Thoracic wound dehiscence	2 (1%)	1 (1%)
Acute abdominal wall dehiscence or hernia	2 (1%)	2 (1%)
Acute diaphragmatic hernia	2 (1%)	0
**Other complications**		
Chyle leak	6 (4%)	9 (5%)
Re-operation for reasons other than bleeding, anastomotic leak, or conduit necrosis	3 (2%)	3 (2%)
**Clavien–Dindo severity classification**		
No complication	63 (39%)	62 (37%)
Grade I	20 (12%)	17 (10%)
Grade II	36 (22%)	44 (26%)
Grade IIIa	20 (12%)	18 (11%)
Grade IIIb	8 (5%)	12 (7%)
Grade IVa	8 (5%)	7 (4%)
Grade IVb	1 (1%)	2 (1%)
Grade V	5 (3%)	4 (2%)

Data are n (%). CDC=US Centers for Disease Control and Prevention.

There were no differences between the groups in baseline HRQOL QLQ-C30 and QLQ-OES 18 questionnaire assessments (appendix p 8). Following neoadjuvant therapy, global health status and physical, role, and emotional functioning deteriorated in both groups, but the magnitude was significantly (p<0·05) greater in the trimodality therapy group, as were symptom scores including fatigue, pain, appetite loss, dyspnoea, and trouble coughing (appendix p 9). At the 1-year follow-up, emotional functioning, pain, and coughing were significantly (p<0·05) worse in the trimodality therapy group than in the perioperative chemotherapy group, with trouble with coughing persisting at 3 years, whereas diarrhoea was significantly (p<0·05) more marked at 1 year and 3 years in the perioperative chemotherapy group.

**Figure 3:**
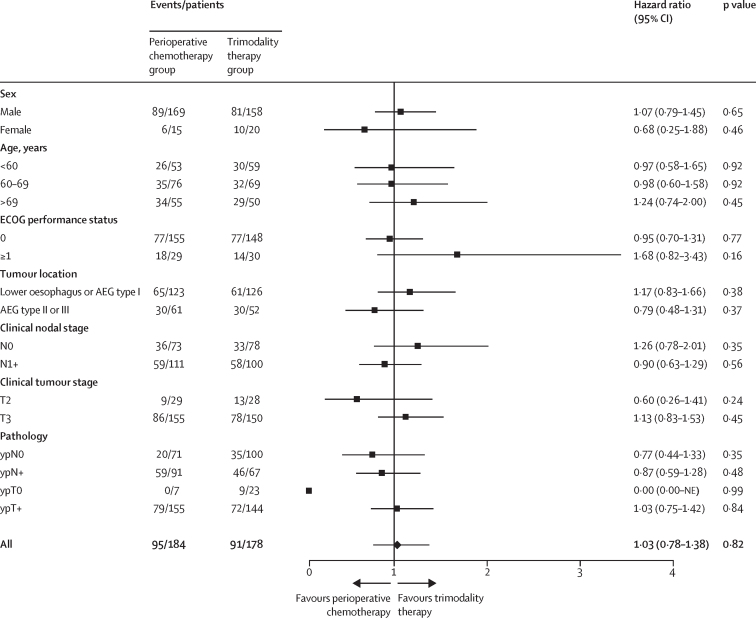
Comparison of survival by baseline characteristics and tumour staging and pathology subgroups AEG=adenocarcinoma of the oesophagogastric junction. ECOG=Eastern Cooperative Oncology Group. NE=not evaluable.

The treatment effect on overall survival according to baseline characteristics and postoperative pathology shows no significant differences between treatment groups (figure 3).

## Discussion

Neo-AEGIS was designed in the context of the CROSS trial, which showed doubling of overall survival with trimodality therapy compared with surgery alone. Uniquely, Neo-AEGIS mandated initial staging with [^18^F]FDG-PET-CT, radiotherapy quality assurance, and strictly defined reporting of operative complications. With a median follow-up of more than 3 years, no significant difference (HR 1·03 [95% CI 0·77–1·38]) was observed in overall survival, with an estimated 3-year survival of 57% with trimodality therapy and 55% with perioperative chemotherapy, and a median survival of 49·2 months with trimodality therapy and 48·0 months with perioperative chemotherapy. These similar survival metrics occurred despite significantly fewer pathological complete and major responses and lower R0 rates in the perioperative chemotherapy group than in the trimodality therapy group. The pattern of failure was not significantly different between treatment groups, and there was no difference in postoperative mortality or major morbidity.

Neo-AEGIS did not complete recruitment, randomly assigning 70% of the target for non-inferiority analysis. In December, 2020, during the COVID-19 pandemic, the data safety monitoring board assessment of the second futility analysis, established a priori for 50% of predicted deaths, was that although futility was not evident, the survival metrics were similar to the first futility analysis conducted at 25% of predicted deaths, and these data were deemed to be unlikely to change with increased numbers and longer-term follow-up. At trial closure, the upper bound of the prespecified 95% CI of 1·16 was exceeded, at 1·38, and non-inferiority was not established. Nonetheless, from a clinical perspective, these data represent the largest randomised series comparing trimodality therapy and perioperative chemotherapy for adenocarcinoma of the oesophagus and oesophagogastric junction, and both primary and secondary endpoints inform a scant literature. At a median follow-up of longer than 3 years, the overall and disease-free survival data appear to be robust and consistent with a conclusion that supports continued equipoise in decision making, in particular for a cancer for which up to 80% of recurrences occur within 2 years.^3^, ^4^ The only comparable phase 3 randomised trial with more than 100 patients was the POET trial, in which 119 patients with endoscopic ultrasound-staged (T3–4, Nx, M0) adenocarcinoma of the oesophagus and oesophagogastric junction received either preoperative chemotherapy (cisplatin plus fluorouracil and leucovorin) or induction chemotherapy followed by chemoradiotherapy (cisplatin and etoposide plus 30 Gy radiotherapy).^12^ The trial closed prematurely due to slow accrual and reported a 3-year survival of 47·4% compared with 27·7% in the chemotherapy group (p=0·07). NeoRes, a phase 2 randomised controlled trial powered on the pathological complete response rate, randomly assigned 181 patients, of whom 131 had adenocarcinoma of the oesophagus and oesophagogastric junction, to three cycles of cisplatin plus flurouracil preoperatively or the same regimen with 40 Gy radiotherapy and reported a 3-year survival rate of 47% for trimodality therapy versus 49% for chemotherapy (p=0·77).^11^

The similar survival outcomes between the two treatment approaches in our trial occurred despite significantly lower pathological response rates and R0 resections in the perioperative chemotherapy group than in the trimodality therapy group. The explanation for the seeming disconnect between gains in pathological complete response and R0 rates and improvement in overall survival remains unclear. The modest observed overall rate of pathological complete response with trimodality therapy (12%) and the differential for perioperative chemotherapy (4%) could be insufficient to affect survival. This finding is consistent with NeoRes and POET, in which higher pathological complete response rates in trimodality groups did not translate to survival benefit.^11^, ^12^ However, more marked differences in major pathological responses (39% *vs* 12%) and R0 rates (96% *vs* 82%) were also observed, as well as apparent nodal downstaging. These findings suggest that modern-era radical surgery was an equalising factor that limited any potential added locoregional benefit of the trimodality approach, and similar locoregional failure rates observed in both groups might be consistent with this hypothesis. A further counterintuitive finding was the similar rates of systemic failure in both groups, notwithstanding significantly reduced liver (p=0·035) and lung (p=0·044) metastases in the perioperative chemotherapy group, suggesting an overall modest effect of the predominant modified MAGIC regimen in this cohort, while acknowledging that the specific impact of FLOT cannot be analysed in a small cohort.^10^, ^23^ Although a cautious interpretation is required given the marked lack of power and wide CIs, forest plots weakly suggest a trend for survival advantage for trimodality therapy in clinical T3 disease, clinical N0 disease, and patients with poorer performance status, whereas perioperative chemotherapy seemed to have an advantage for patients with clinical node-positive disease, T2, pathological node-negative disease, and a complete response at the primary site (ypT0). This latter finding might be consistent with a cohort study report of superior recurrence-free survival in patients with a pathological complete response after chemotherapy compared with those with a pathological complete response after chemoradiotherapy, presenting a hypothesis that requires further evaluation.^23^

With no differences in overall or disease-free survival between treatment groups, secondary endpoints including postoperative mortality and morbidity, toxicity and tolerance, and HRQOL might inform clinician and patient choices.^11^, ^12^, ^13^ The potential added risk of preoperative chemoradiotherapy is of particular interest, as oesophageal cancer surgery is associated with a risk of postoperative mortality greater than any other elective cancer operation and has been a major point in centralisation debates and policies internationally.^24^ Operative mortality rate was 6·5% with trimodality therapy versus 2·6% with chemotherapy in NeoReS, and 10·2% versus 3·8% in POET, with deaths particularly related to major pulmonary complications.^11^, ^12^ By contrast, in Neo-AEGIS, 90-day mortality was not significantly different at 2% with trimodality therapy versus 3% with perioperative chemotherapy, both below the modern international benchmark of 4·5%.^25^ Moreover, with strictly defined reporting of operative complications used in an upper gastrointestinal randomised trial for the first time, key index complications including pneumonia, respiratory failure, and anastomotic leaks were observed at similar rates, as was the severity of complications. The establishment of radiotherapy quality assurance within the trial design, with particular focus on planning treatment volumes, and doses to organs at risk in particular lungs, might be relevant to operative outcomes, particularly for true thoracic tumours including adenocarcinoma of the oesophagus and AEG type I, which were most common in this trial population. Accordingly, the interpretation of both operative and oncological outcomes in Neo-AEGIS should be viewed in the context of targeting modern optimum standards of care in specialist centres.

The toxicity profile and tolerance of the entire prescribed regimen showed anticipated differences between treatment groups. The most frequent grade 3 or 4 adverse event with perioperative chemotherapy was neutropenia (in 49 [27%] of 183 patients). This frequency was less than in the FLOT4 trial, at 40% for the modified MAGIC regimen and 50% for FLOT, perhaps reflecting a greater use of granulocyte colony-stimulating factor in Neo-AEGIS.^10^ Just 41% of patients could tolerate all cycles of chemotherapy, consistent with the less than 50% observed in the FLOT4 and MAGIC trials.^6^, ^10^ Moreover, seven (4%) patients receiving perioperative chemotherapy, compared with two (1%) receiving trimodality therapy, did not progress to surgery due to deteriorated health. Conversely, trimodality therapy had a significantly greater effect on HRQOL after treatment and before surgery. However, by 1 year and 3 years of follow-up, HRQOL differences had largely equalised, with clinically relevant changes, defined as greater than 10 points difference, only persisting for coughing in the trimodality therapy group. This finding is consistent with the HRQOL analysis of NeoRes, with coughing significantly worse in long-term follow-up in the trimodality group; however, this effect was not observed in the CROSS trial HRQOL follow-up report.^26^, ^27^

The principal limitation of Neo-AEGIS is that at its termination it did not provide statistical proof to underpin conclusions. Of relevance to the initial power calculation, and subsequent revisions, the 3-year survival of 55% for the perioperative chemotherapy group markedly exceeded the initial projection of 43%, perhaps reflecting that myriad factors affect survival outcomes in patients with adenocarcinoma of the oesophagus and oesophagogastric junction in the modern era. In addition, the FLOT4 trial data, published in 2019, and its immediate impact on management trends for gastric and adenocarcinoma of the oesophagus and oesophagogastric junction, was not predictable. Although these randomised trials are distinct, it is notable that the median and 3-year survival outcomes for the perioperative chemotherapy group in Neo-AEGIS, at 48 months and 55%, respectively, is markedly superior to the control group (ECF or ECX) in the FLOT4 trial, at 35 months and 48%, and similar to the FLOT treatment group, at 50 months and 57%.^10^ Finally, the COVID-19 pandemic presented an existential barrier to the conduct of clinical trials internationally and was relevant to the decision to terminate the trial.

If continued clinical equipoise is a valid interpretation, how does this weigh up for proponents of each approach? For the group that received trimodality therapy used in the CROSS trial, the clinical and survival outcomes were similar to the CROSS trial, with no increase in major operative morbidity or mortality. A markedly lower pathological complete response rate was, however, observed, at 12% versus 23%. With radiotherapy quality-assured in Neo-AEGIS, this finding cannot be explained but most plausibly suggests differences in primary tumour extent. 10-year outcome data from the CROSS trial show a 36% survival rate and highlight reduced locoregional recurrence; however, the absence of an effect on systemic relapse revealed the limitations of this approach.^28^ In this context, adjuvant immunotherapy holds promise. The CheckMate 577 trial evaluated nivolumab as adjuvant therapy after trimodality therapy in patients with residual pathological disease and an R0 resection after chemoradiotherapy and reported a median disease-free survival of 22 months versus 11 months with placebo; the overall survival data are eagerly awaited.^29^ For perioperative chemotherapy, predominantly with a modified MAGIC regimen, Neo-AEGIS suggests that combination chemotherapy and radiotherapy might not be required in locally advanced (predominantly clinical T3N0–1) adenocarcinoma of the oesophagus and oesophagogastric junction, with the imperative being to ensure the provision of high-quality surgery. Moreover, the toxicity of regimens used were acceptable, as were HRQOL data, and the preoperative duration of therapy is shorter than for the trimodality regimen. However, the prospect of postoperative chemotherapy, and the fact that fewer than 50% of patients completed the prescribed regimen, might influence clinician and patient choice. The size of the FLOT subset (27 [15%] of 184 participants) precludes subset analysis, and we acknowledge that if evaluable it might have resulted in a greater biological effect at both primary and metastatic sites compared with the modified MAGIC regimen.^10^, ^23^ The results of ESOPEC (NCT02509286) will be informative. This trial directly compares trimodality therapy with the FLOT regimen in patients with adenocarcinoma of the oesophagus and oesophagogastric junction, with similar inclusion criteria to Neo-AEGIS.^30^ It is powered on FLOT being 13% superior to the CROSS trimodality regimen in 3-year survival (HR 0·65) and will be of major interest alone and as a companion and comparison to Neo-AEGIS.^30^ In addition, the narrative of the debate on the optimum approach to treat locally advanced adenocarcinoma of the oesophagus and oesophagogastric junction will evolve in the years ahead, informed by studies of neoadjuvant and adjuvant immunotherapy combined with chemotherapy, including MATTERHORN (NCT04592913), Keynote-585 (NCT03221426), and DANTE (NCT03421288).

In conclusion, perioperative chemotherapy shows similar survival metrics to the CROSS trimodality regimen despite inferior pathological responses and surgical margin rates. No added operative risk from trimodality therapy was evident, and HRQOL outcomes were similar. Although Neo-AEGIS was underpowered for the assessment of non-inferiority, and futility was not observed, we believe that these comprehensive data provide modern benchmarks of oncological, operative, and HRQOL outcomes and could inform practice and decision making. We encourage continued clinical equipoise in treatment selection and suggest at this time that factors including patient choice, the logistics of combining chemotherapy, radiotherapy, and surgery, which vary from country to country, and access to adjuvant immunotherapy, might be of practical and pragmatic importance in the curative approach to a complex cancer.

For the **protocol** see https://www.cancertrials.ie/wp-content/uploads/2023/09/CTRIAL-IE-10-14-Protocol-Version-12_dated-27-Jul-20-and-appendices.pdf

## Data sharing

Cancer Trials Ireland supports the wider dissemination of information from the research it conducts and increased cooperation between investigators. Trial data are collected, managed, stored, shared, and archived according to Cancer Trials Ireland and Health Research Board Clinical Research Facility Standard Operating Procedures to ensure the enduring quality, integrity, and utility of the data. Formal requests for data sharing are considered in line with Cancer Trials Ireland procedures with due regard given to patient consent. Data requests can be submitted to Cancer Trials Ireland via info@cancertrials.ie and must include a description of the research proposal. Data sharing requests are reviewed and approved by the chief investigator, the trial steering committee, and Cancer Trials Ireland in terms of scientific merit and ethical considerations including patient consent. Data recipients are required to enter a formal data sharing agreement, which describes the conditions for release and requirements for data transfer, storage, archiving, publication, and intellectual property.

## Declaration of interests

SRP reports support for the present manuscript from Cancer Research UK; payment or honoraria for lectures, presentations, speaker bureaus, manuscript writing, or educational events from Jabar Al Ahmed Hospital, Kuwait City, Kuwait; support for attending meetings or travel from the Ministry of Health Kuwait. MAL reports payment or honoraria for lectures, presentations, speaker bureaus, manuscript writing or educational events from AstraZeneca; participation on a data safety monitoring board or advisory board for Servier and Agios; other financial or non-financial interests (educational grant to institution from Roche and being principal investigator on clinical trials for MSD, Basilea, Exilexis, Astellas, Daichii Sancho, and Zymeworks). SC reports support for attending meetings or travel from MSD, Pfizer, and Roche. GOG reports payment or honoraria for lectures, presentations, speaker bureaus, manuscript writing, or educational events from AstraZeneca and AbbVie. IP reports support for the present manuscript from Health Research Board, Irish Cancer Society, and Oesophageal Cancer Fund to Cancer Trials Ireland. RR reports consulting fees from Servier; payment or honoraria for educational events from Bristol-Myers Squibb and Servier; support for attending meetings from Servier Laboratories; and advisory board fees from Servier Laboratories. SF reports payment or honoraria for lectures, presentations, speaker bureaus, manuscript writing, or educational events from Servier. GP reports support for the present manuscript from the French National Cancer Institute; grants or contracts from the French National Cancer Institute; consulting fees from Bristol-Myers Squibb, Astellas, and Nestle; payment or honoraria for lectures, presentations, speaker bureaus, manuscript writing, or educational events from the European Society for Medical Oncology; and support for attending meetings or travel from Metronic. DO'T reports honoraria for lectures and speaker bureaus from Ipsen, Novartis, Wyeth Ledrele, and AstraZeneca; support for attending meetings or travel from Ipsen, Novartis, and AstraZeneca; and unpaid leadership roles in other board, society, committee or advocacy groups (European Neuroendocrine Tumour Society Board). RM reports payment or honoraria for lectures, presentations, speaker bureaus, manuscript writing, or educational events from Bayer, Sanofi, Janssen, MSD, Pfizer, Novartis, Clovis, Astellas, Ipsen, and Bristol-Myers Squibb, and support for attending meetings from Pfizer, Janssen, Roche, and Ipsen. All other authors declare no competing interests.
