# Supplementary material for: Trimodality therapy versus perioperative chemotherapy in the management of locally advanced adenocarcinoma of the oesophagus and oesophagogastric junction (Neo-AEGIS): an open-label, randomised, phase 3 trial
Source: Lancet Gastroenterol Hepatol. 2023 Sep 18;8(11):1015–27. doi: 10.1016/S2468-1253(23)00243-1 (PMC10567579; doi:10.1016/S2468-1253(23)00243-1)
Supplement: Supplementary appendix [file mmc1.pdf]

THE LANCET
Gastroenterology & Hepatology

Supplementary appendix

This appendix formed part of the original submission and has been peer reviewed. We post it as supplied by the authors.

Supplement to: Reynolds JV, Preston SR, O'Neill B, et al. Trimodality therapy versus perioperative chemotherapy in the management of locally advanced adenocarcinoma of the oesophagus and oesophagogastric junction (Neo-AEGIS): an open-label, randomised, phase 3 trial. *Lancet Gastroenterol Hepatol* 2023; published online Sept 18. https://doi.org/10.1016/S2468-1253(23)00243-1.

Neo-AEGIS Investigators and Trial Group

Prof John V Reynolds FRCS, Prof Maeve A Lowery Mb,BCh,BA, Sinead Cuffe MD, Narayanasamy Ravi FRCS, Prof Dermot O'Toole MD, Ciaran Johnston FFR RCSI, St James's Hospital, Dublin, Ireland.

Moya Cunningham MD, Brian O'Neill FFR RCSI, St. Luke's Radiation Oncology Network, Dublin, Ireland.

Lene Baeksgaard MD, Signe Lenora Risumlund MD, Michael P Achiam D.M.Sc, Rigshopitalet, Copenhagen, Denmark.

Rajarshi Roy FRCR, Hull University Teaching Hospitals NHS Trust, Castle Hill Hospital, Castle Road, Cottingham, HU16 5JQ, United Kingdom.

Stephen Falk MD, University Hospitals Bristol and Weston NHS Foundation Trust, Bristol Haematology and Oncology Centre, Bristol, BS2 8ED, United Kingdom.

Prof George B Hanna Ph.D, St. Mary's Hospital, Imperial College Healthcare NHS Trust, Praed Street, London, W2 1NY, United Kingdom.

Frederick R Bartlett MD(Res), Portsmouth Hospitals University NHS Trust, Queen Alexandra Hospital, Cosham, Portsmouth, PO6 3LY, United Kingdom.

Shaun R Preston MD, Royal Surrey County Hospital NHS Foundation Trust, Egerton Road, Guildford, Surrey, GU2 7ZZ, United Kingdom.

Richard C Turkington MD, Belfast Health and Social Care Trust, Northern Ireland Cancer Centre, Belfast City Hospital, Lisburn Road, Belfast, BT9 7AB, United Kingdom.

Shajahan Wahed MD, Northern Oesophago-Gastric Unit, Royal Victoria Infirmary, Newcastle upon Tyne, United Kingdom.

Sharmila Sothi MBBS, University Hospitals Coventry & Warwickshire, Clifford Bridge Road, Walsgrave, Coventry, CV2 2DX, United Kingdom.

Hugo Ford MD, Cambridge University Hospitals NHS Foundation Trust, Hills Rd, Cambridge, CB2 0QQ, United Kingdom

Martin S Wadley MD, Worcestershire Acute Hospitals NHS Trust, Worcestershire Oncology Centre, Worcestershire Royal Hospital, Charles Hastings Way, Worcester, WR5 1DD, United Kingdom.

Dr Somnath Mukherjee FRCS, Oxford University Hospital NHS Trust Churchill Hospital, Oxford Cancer Centre, Old Road, Headington, Oxford, OX3 7LE, United Kingdom.

Derek Power MD, Cork University Hospital and Mercy University Hospital, Cork, Ireland.

Carys Morgan FRCR, Tom Crosby FRCR, Velindre University NHS Trust, Unit 2, Charnwood Court, Cardiff, United Kingdom.

Simon L Parsons DM FRCS, Nottingham City Hospital, Nottingham University Hospitals NHS Trust, Hucknall Road, Nottingham, NG5 1PB, United Kingdom.

Prof Guillaume Piessen MD, Claude Huriez University Hospital, Lille, France.

Neel Bhuva FRCP, Mount Vernon Cancer Centre, East and North Hertfordshire NHS Trust, Rickmansworth Road, Northwood, Middlesex, HA6 2RN, United Kingdom.

Sorcha Campbell MBChB, NHS Lothian, Edinburgh Cancer Centre, The Western General Hospital, Edinburgh, EH4 2XU, United Kingdom.

Prof Magnus Nilsson Ph.D, Division of Surgery, CLINTEC, Karolinska Institutet and Department of Upper Abdominal Diseases, Karolinska University Hospital, Stockholm, Sweden.

Prof Liam Grogan MD, Beaumont Hospital, Beaumont Road, Dublin 9, Ireland.

Greg Leonard MB BCH BAO, University Hospital Galway, Newcastle Road, Galway, Ireland.

Andrew R Bateman Ph.D, University Hospital Southampton NHS Foundation Trust, Southampton General Hospital, Tremona Road, Southampton, SO16 6YD, United Kingdom.

Catherine Mitchell FRCR, Royal Preston Hospital, Sharoe Green Lane, Fulwood, Preston, PR2 9HT, United Kingdom.

Prof Raymond S. McDermott MD, Prof Seamus O'Reilly MD, Eibhlin Mulroe MBA, Olivia McLoughlin B.Sc, Imelda Parker Ph.D, Anna Shevlin M.Sc, Aoife M Shannon Ph.D, Jacinta Marron B.Sc, Marc Nolan B.Sc, Grace Burch B.Sc, Cancer Trials Ireland, Dublin 2, Ireland

Alberto Alvarez-Iglesias Ph.D, Michelle Costello B.Sc, HRB Clinical Research Facility, NUI Galway, Galway, Ireland

Gareth O Griffiths Ph.D, Daniel Griffiths B.Sc, Kelly Cozens M.Sc, Southampton Clinical Trials Unit, University of Southampton, Southampton, United Kingdom

Emma Foley M.Sc, Claire L Donohoe Ph.D, Catherine O'Farrell M.Sc, Jennifer Moore H.Dip (oncology nursing), St James's Hospital, Dublin 8, Ireland

Prof Prof Jacintha O'Sullivan Ph.D, Trinity Translational Medicine Institute, Trinity Centre for Health Sciences, St James Hospital, Dublin 8, Ireland.

Trial Sites and Accrual

Prof John V Reynolds FRCS, St James's Hospital, Dublin, Ireland in collaboration with Moya Cunningham MD St. Luke's Radiation Oncology Network, Dublin, Ireland. 125 patients

Lene Baeksgaard MD, Rigshopitalet, Copenhagen, Denmark. 38 patients.

Rajarshi Roy FRCR, Hull University Teaching Hospitals NHS Trust, Castle Hill Hospital, Castle Road, Cottingham, HU16 5JQ, United Kingdom. 32 patients.

Stephen Falk MD, University Hospitals Bristol and Weston NHS Foundation Trust, Bristol Haematology and Oncology Centre, Bristol, BS2 8ED United Kingdom. 24 patients.

Prof George B Hanna Ph.D, St. Mary's Hospital, Imperial College Healthcare NHST Trust, Praed Street, London, W2 1NY, United Kingdom. 17 patients.

Frederick R Bartlett MD(Res), Portsmouth Hospitals University NHS Trust, Queen Alexandra Hospital, Cosham, Portsmouth, PO6 3LY, United Kingdom. 17 patients.

Shaun R Preston MD, Royal Surrey County Hospital NHS Foundation Trust, Egerton Road, Guildford, Surrey, GU2 7ZZ, United Kingdom. 15 patients

Richard C Turkington MD, Belfast Health and Social Care Trust, Northern Ireland Cancer Centre, Belfast City Hospital, Lisburn Road, Belfast, BT9 7AB United Kingdom. 14 patients.

Shajahan Wahed MD, Cumbria, Northumberland, Tyne and Wear NHS Foundation Trust, Northern Oesophago-Gastric Unit, Royal Victoria Infirmary, Queens Victoria Road, Newcastle upon Tyne, NE1 4LP, United Kingdom. 14 patients.

Sharmila Sothi MBBS, University Hospitals Coventry & Warwickshire, Clifford Bridge Road, Walsgrave, Coventry, CV2 2DX, United Kingdom. 12 patients.

Hugo Ford MD, Cambridge University Hospitals NHS Foundation Trust, Hills Rd, Cambridge, CB2 0QQ, United Kingdom. 12 patients.

Martin S Wadley MD, Worcestershire Acute Hospitals NHS Trust, Worcestershire Oncology Centre, Worcestershire Royal Hospital, Charles Hastings Way, Worcester, WR5 1DD, United Kingdom. 12 patients.

Dr Somnath Mukherjee FRCS, Oxford University Hospital NHS Trust Churchill Hospital, Oxford Cancer Centre, Old Road, Headington, Oxford, OX3 7LE, United Kingdom. 8 patients.

Derek Power MD, Cork University Hospital and Mercy University Hospital, Cork, Ireland. 7 patients.

Carys Morgan FRCR, Velindre University NHS Trust, Unit 2, Charnwood Court, Cardiff, United Kingdom. 7 patients.

Simon L Parsons DM FRCS, Nottingham City Hospital, Nottingham University Hospitals NHS Trust, Hucknall Road, Nottingham, NG5 1PB, United Kingdom. 6 patients.

Prof Guillaume Piessen MD, Claude Huriez University Hospital, Lille, France. 4 patients.

Neel Bhuva FRCP, Mount Vernon Cancer Centre, East and North Hertfordshire NHS Trust, Rickmansworth Road, Northwood, Middlesex, HA6 2RN, United Kingdom. 4 patients.

Sorcha Campbell MBChB, NHS Lothian, Edinburgh Cancer Centre, The Western General Hospital, Edinburgh, EH4 2XU, United Kingdom. 3 patients.

Prof Magnus Nilsson Ph.D, Division of Surgery, CLINTEC, Karolinska Institutet and Department of Upper Abdominal Diseases, Karolinska University Hospital, Stockholm, Sweden. 2 patients.

Prof Liam Grogan MD, Beaumont Hospital, Beaumont Road, Dublin 9, Ireland. 1 patient.

Greg Leonard MB BCH BAO, University Hospital Galway, Newcastle Road, Galway, Ireland. 1 patient.

Andrew R Bateman Ph.D, University Hospital Southampton NHS Foundation Trust, Southampton General Hospital, Tremona Road, Southampton, SO16 6YD, United Kingdom. 1 patient.

Catherine Mitchell FRCR, Royal Preston Hospital, Sharoe Green Lane, Fulwood, Preston, PR2 9HT, United Kingdom. 1 patient.

Statistical Design and Evolution within the Trial and Basis for Trial Closure

Initial Trial Design 2013-2017

The study primary end-point is overall survival of patients treated pre and postoperative chemotherapy (perioperative chemotherapy) versus neoadjuvant chemoradiotherapy followed by surgery (trimodality). Originally, in 2013 the study was designed with a two-sided alpha level of 0·05, and an estimated 80% power to detect a 3-year increase in overall survival of 15% (58% vs 43%) through the use of the CROSS protocol, with a sample size of 366 patients. It was modified to 10% in 2014 following agreement between Cancer Trials Ireland (CTI) and the UK National Cancer Research Institute (NCRI), and powered to detect a 3-year increase in overall survival of 10% (53% vs 43%) through the use of the CROSS protocol, and with initial evaluation at 3 years of follow up of the last patient. A two-sided log rank test with an overall sample size of 594 subjects (297 in each treatment arm) was estimated, with the 10% difference to achieve 80% power at a 0·05 significance level corresponding to a hazard ratio (HR) of 1·33. The data was to be analysed three years after the last patient entered the study. The proportion of non-evaluable patients was assumed to be 10%, and the results would be based on the assumption that the hazard rates (HR) are proportional.

Non-inferiority Protocol Amendment, approved May 2018 (Version 10·2)

The statistical considerations and sample size were updated following the addition of the FLOT regimen as an option to Arm A. A total of 628 patients (314 patients per treatment arm) was required to provide 80% power to test the non-inferiority of MAGIC/FLOT versus CROSS, assuming the expected 3-year OS for both arms is 53%, and using a non-inferiority margin of 8·5% (corresponding to a non-inferiority HR of 1·275 where MAGIC/FLOT is the numerator) and an alpha level of 5%. The data would be analysed three years after the last patient enters the trial. The period over which patients were to be recruited is considered to be 6 years for the purposes of the sample size calculation, and the proportion of patients lost during follow-up was assumed to be 5%. These calculations were based on the exponential survival function and the assumption that the hazard rates are proportional.

Sample size adjustment after first futility analysis, December 2018, at n=76 deaths

The first futility analysis provided 3 year survival estimates of 52% for CROSS, and 57% for MAGIC. The HR was 0·98 (ratio calculated as Arm A / Arm B) with a 95% confidence interval of 0·62 to 1·53. The non-inferiority limit of 1·275 is included in the interval and therefore there is no evidence at this stage to declare the modified MAGIC regimen unacceptably inferior when compared to the CROSS protocol. The analysis revealed that the '…the estimated 3-year survival rate of 57% for the modified MAGIC regimen is higher than the survival rate of 48% used in the sample size calculation in the current protocol' which was based on the results of the FLOT 4 trial. Hence the sample size was recalculated to 540 which was approved by the DSMB in December 2018, and approved in Protocol V11 in June 2019. A total of 540 patients (270 patients per treatment arm) would be required to provide 80% power to test the non-inferiority of MAGIC/FLOT vs CROSS. This assumed an expected 3-year OS rates are 57% and 53% respectively, and uses a non-inferiority margin of 5% (corresponding to a non-inferiority HR of 1·16 where MAGIC/FLOT is the numerator) and an alpha level of 5%. The data would be analysed three years after the last patient enters the study trial. The period over which patients were to be recruited was considered to be six years for the purposes of the sample size calculation, and the proportion of patients lost during follow-up assumed to be 5%.

Second futility analysis at 50% of predicted deaths (n=143), November 2020

The 3-year estimated survival probability for the MAGIC/FLOT was 57% (95% CI 48% to 65%) and for the CROSS arm it is 56% (95% CI 47% to 64%). The HR was 1·02 (ratio calculated as Arm A/Arm B) with a 95% confidence interval of 0·74 to 1·42. The non-inferiority limit of 1 is included in the interval and therefore there was no evidence at this stage to declare the MAGIC/FLOT regimen as unacceptably inferior when compared to the CROSS protocol. These estimated probabilities are in close agreement with those assumed in the sample size calculations (57% and 53% for MAGIC/FLOT and CROSS, respectively), and would not indicate that the sample size calculation should be re-visited. The 3-year estimated survival probability for the MAGIC regimen of 57% is notably the same as the first futility analysis. The data for the FLOT regime is too early (patients have been followed for overall survival for a maximum of 2 years), so the 3-year survival probability cannot be estimated.

Trial Closure to Recruitment in December 2020

Trial closure in December 2020 followed the DSMB assessment of the second futility analysis, where similar survival probability to the first futility analysis and similar survival metrics in both study arms was observed. In addition, continued problems of recruitment during the first 9 months of the COVID-19 pandemic and future uncertainty in this context, as well as the potential emerging impact of adjuvant immunotherapy on practice, were factors cited by the DSMB. The final data analysis was in March 2023.

Trial Treatment and Monitoring

CROSS

CROSS consisted of chemotherapy and radiotherapy run concurrently over a four and a half-week period. Chemotherapy was administered for five preoperative cycles. Each 1-week cycle consisted of Paclitaxel 50 mg/m² and carboplatin AUC 2mg/ml/min intravenously (IV) on day 1. Radiation therapy (41.4 Gy/23 fractions) was administered for 4.5 weeks. The radiation commenced on the 1st day of treatment and ran as follows: days 1-5, days 8-12, days 15-19, days 22-26 and days 29-31 inclusive.

MAGIC or FLOT

Decisions about which chemotherapy regimen to use, i.e. modified MAGIC or FLOT, were made by the treating clinician.

Modified MAGIC (EO(C)X(F)) regimen is a combination of epirubicin, cisplatin or oxaliplatin and a choice of 5-fluorouracil or capecitabine. The choice between administering cisplatin or oxaliplatin and 5-fluorouracil or capecitabine was at the discretion of the investigator. MAGIC was administered for three preoperative cycles followed by three postoperative cycles. Each three week cycle of MAGIC consisted of epirubicin 50 mg/m² IV on day 1, (cisplatin 60 mg/m² IV on day 1 or oxaliplatin 130 mg/m2 IV on day 1), and (fluorouracil 200 mg/m² as continuous intravenous infusion on days 1 to 21 or capecitabine 625 mg/m2 administered orally twice daily on days 1 to 21).

FLOT was administered for four preoperative cycles followed by four postoperative cycles. Each two week cycle of FLOT consisted of docetaxel 50 mg/m² IV on day 1, oxaliplatin 85 mg/m² IV on day 1, leucovorin 200 mg/m² IV on day 1, and 5-FU 2600 mg/m² as 24-h infusion on day 1.

Granulocyte colony stimulating factor (G-CSF) was permitted as prophylaxis. Dose modification schedule and the recommended supportive therapy were given per protocol: https://www.cancertrials.ie/wp-content/uploads/2023/07/CTRIAL-IE-10-14-Protocol-Version-12_dated-27-Jul-20_Final-Clean.pdf

Therapy was stopped prematurely for unacceptable toxicity, disease progression, death, or at the patient's request.

Surgery was scheduled 3-10 weeks after the last dose of preoperative chemotherapy.

Frequency of Assessments.

Patients were assessed according to medical history, physical examination, vitals, weight, ECOG performance status, American Society of Anesthesiologists (ASA) grading system, complete blood count, biochemistry blood tests, CT-PET, Endoscopic Ultrasound, Oesophago-gastro-duodenoscopy (OGD), electrocardiogram, MUGA or ECHO, pulmonary function testing, pregnancy test (if applicable) and health-related quality of life (HR-QL), at baseline.

Patients were assessed for physical examination, vitals, weight, ECOG performance status and bloods tests before the start of every cycle. Restaging by means of PET-CT or CT, OGD was done within 4-8 weeks of finishing treatment. Follow-up included physical examination, weight, ECOG performance status, bloods tests and HR-QL 4-8 weeks post treatment, three monthly post–surgery for the first year and six monthly thereafter for five years or until disease progression or death. Follow-up included annual PET-CT or CT, OGD for three years or until disease progression, recurrence or death. Post disease progression or five years standard follow-up, patients were followed up for survival every six months until death or until the end of the trial, whichever occurs earlier.

Neo-AEGIS Inclusion and Exclusion Criteria

Inclusion Criteria

1. Histologically verified adenocarcinoma of the oesophagus, or oesophago-gastric junction based on Oesophago-gastro-duodenoscopy.
2. CT-FDG-PET performed in all patients for disease staging
3. Endoscopic Ultrasound in all patients unless luminal obstruction precludes sensitivity of the test.
4. Staging laparoscopy will be performed at the investigator's discretion for locally advanced AEG II and AEG III tumours.
5. Pre-treatment stage cT2-3, N0-3, M0.
6. Maximum tumour length should be no more than 8cm (equal to 8 cm is acceptable).
7. Male/female patients aged ≥18 years.
8. ECOG Performance Status 0, 1 or 2
9. American Society of Anesthesiologists Grading I-II
10. Adequate cardiac function. For all patients, an ejection fraction of > 50% is required. If patients have a known history of significant cardiac disease (e.g. known ischemic disease, cardiomyopathy) an ejection fraction > 50% and cardiac clearance by a consultant cardiologist for major surgery and cancer therapies is required.
11. Adequate respiratory function. Patients should have pulmonary function tests completed with a minimum Forced expiratory Volume (FEV1) ≥1.5L. Cardio-Pulmonary exercise testing acceptable.
12. Adequate bone marrow function: absolute neutrophil count >$1.5x10^9$/l; white blood cell count > $3x10^9$/l; platelets > $100x10^9$/l; haemoglobin > 9g/dl (can be post-transfusion).
13. Adequate renal function: glomerular filtration rate > 60ml/minute calculated using the Cockcroft-Gault Formula
14. Adequate liver function: serum bilirubin ≤ ULN; AST <2.5x ULN and ALP <3x ULN (ULN as per institutional standard).
15. Written informed consent must be obtained from the patient before any study-specific procedures are performed.
16. Women of child-bearing potential and male subjects must agree to use an effective barrier method of contraception for up to 6 months following discontinuation of therapy. Effective barrier method of contraception is defined as any medically recommended (or combination of methods) as per standard of care.
17. Women of child bearing potential must have pregnancy excluded by urine or serum beta-HCG testing within 7 calendar days prior to registration.

Exclusion Criteria

1. Tumours of squamous histology.
2. Patients with advanced inoperable or metastatic oesophageal, junctional or gastric adenocarcinoma.
3. Disease length (total length of tumour plus node) greater than 10 cm (up to 10 cm will be allowed) -*measured by any modality or, if appropriate, combination of modalities*-, unless in the opinion of the investigator in discussion with national radiotherapy lead, it is felt that organs at risk constraints are likely to be achievable.
4. Any prior chemotherapy for gastrointestinal cancer.
5. Prior abdominal, thoracic, chest wall or breast radiotherapy.
6. Patients who are unfit for surgery or cancer treatments based on cardiac disease.
7. Patients with acute systemic infections.
8. Patients who are receiving treatment with sorivudine or its chemical related analogues, such as brivudine which is contraindicated with capecitabine and 5-fluorouracil administration.
9. Clinical Chronic Obstructive Pulmonary Disease with significant obstructive airways disease classified by FEV1 < 1.5 L or PaO_2 less than 9kPa on room air.
10. Known peripheral neuropathy >Grade 1 (absence of deep tendon reflexes as the sole neurological abnormality does not render the patient ineligible).
11. Known positive tests for human immunodeficiency virus infection, acute or chronic active hepatitis B infection.
12. Any other malignancies within the last 5 years (other than curatively treated basal cell carcinoma of the skin and/or in situ carcinoma of the cervix).
13. Participation in other clinical trials of investigational or marketed agents for the treatment of oesophageal cancer or other diseases within 30 days prior to registration. UK sites please refer to Group Specific Appendix.
14. Women who are pregnant or breastfeeding.
15. Psychiatric illness/social situations that would limit compliance with study requirements.
16. Known Dihydropyrimidine dehydrogenase deficiency

For sites in UK, France, Denmark and Sweden: for inclusion and exclusion criteria please also refer to the applicable Country Specific Appendix to the protocol https://www.cancertrials.ie/wp-content/uploads/2023/09/CTRIAL-IE-10-14-Protocol-Version-12_dated-27-Jul-20-and-appendices.pdf

Supplemental Table 1: Health-related quality of life at Baseline, Preoperative and 1 and 3 years postoperatively (Mean (95% CI)

	Baseline		Preoperative		1 year post-op		3 year post-op	
	MAGIC/FLOT n = 182	CROSS n = 176	MAGIC/FLOT n = 163	CROSS n = 169	MAGIC/FLOT n = 94	CROSS n = 97	MAGIC/FLOT n = 44	CROSS n = 45
**Overall HR-QL**								
**Global health status**	78 (75,81)	80 (77,82)	78 (75,82)	72 (69,76)	72 (68,77)	73 (68,77)	77 (71,83)	76 (70,82)
**QLQ-C30 Functional scales**								
**Physical functioning**	95 (94,97)	95 (93,96)	91 (89,93)	87 (85,90)	87 (84,91)	85 (81,89)	87 (80,93)	87 (82,93)
**Role functioning**	89 (86,92)	92 (89,94)	82 (78,87)	78 (73,83)	79 (73,85)	78 (72,85)	85 (77,94)	82 (74,91)
**Emotional functioning**	82 (79,85)	82 (79,85)	85 (82,88)	81 (78,84)	89 (86,92)	82 (77,87)	84 (78,90)	84 (77,90)
**Cognitive functioning**	93(91,95)	93 (91,95)	89 (86,92)	89 (86,92)	86 (82,90)	88 (84,93)	83 (77,89)	84 (77,91)
**Social functioning**	86 (83,90)	88 (85,91)	82 (77,86)	79 (75,83)	82 (77,88)	83 (78,89)	85 (76,93)	87 (80,94)
**QLQ-C30 Symptom Scales**								
**Fatigue**	15 (12,17)	15 (13,18)	21 (18,25)	28 (24,32)	29 (24,34)	27 (21,32)	28 (21,34)	27 (18,35)
**Nausea and vomiting**	9 (6,11)	8 (6,10)	8 (5,11)	9 (6,12)	10 (6,14)	11 (7,14)	12 (5,18)	9 (4,14)
**Pain**	15(12,18)	12 (9,15)	8 (5,11)	14 (10,17)	17 (11,22)	22 (16,28)	17 (9,25)	14 (7,20)
**Dyspnoea**	3 (2,5)	5 (3,7)	11 (7,14)	13 (10,17)	16 (11,21)	21 (16,27)	23 (14,32)	21 (12,29)
**Insomnia**	20 (17,24)	22 (18,26)	20 (15,25)	18 (14,23)	26 (19,33)	22 (16,29)	27 (18,36)	21 (12,29)
**Appetite loss**	22 (18,27)	18 (14,22)	11 (7,14)	19 (14,23)	17 (12,22)	19 (13,25)	13 (6,21)	12 (5,20)
**Constipation**	15 (11,19)	14 (10,17)	8 (5,11)	13 (9,17)	9 (5,13)	10 (6,14)	9 (2,16)	12 (5,19)
**Diarrhoea**	5 (3,7)	6(3,8)	5 (3,7)	6 (4,9)	20 (16,25)	15 (10,21)	12 (7,18)	11 (3,18)
**Financial difficulties**	14 (10,19)	9 (6,13)	16 (11,21)	15 (11,20)	15 (9,21)	16 (10,23)	11 (4,19)	13 (4,22)
**QLQ- OES 18**								
**Eating difficulties**	32 (28,36)	27 (23,30)	15 (11,19)	17 (13,20)	22 (17,26)	24 (20,29)	17 (11,23)	21 (14,29)
**Reflux**	15 (12,18)	12 (9,15)	12 (8,16)	10 (7,13)	24 (19,30)	21 (16,26)	24 (15,33)	27 (17,36)
**Oesophageal pain**	17 (14,20)	17 (14,20)	8 (5,10)	12 (9,15)	12 (9,16)	16 (11,21)	14 (7,20)	14 (8,21)
**Dysphagia**	79 (76,82)	77 (73,81)	88 (84,92)	86 (82,90)	89 (84,93)	87 (82,92)	93 (89,98)	86 (79,94)
**Dry mouth**	20(16,24)	18 (13,22)	22 (18,27)	21 (16,26)	22 (16,28)	16 (10,21)	26 (16,36)	22 (13,31)
**Trouble with taste**	8 (5,11)	7 (4,10)	22 (17,27)	20 (15,26)	11 (6,15)	11 (6,16)	7 (1,13)	11 (3,19)
**Trouble swallowing saliva**	12(8,16)	11 (7,15)	8 (4,12)	5 (2,8)	8 (3,13)	5 (2,8)	6 (0,11)	7 (1,13)
**Choked when swallowing**	10 (7,13)	10 (7,13)	6 (3,9)	5 (2,8)	9 (5,14)	5 (2,8)	6 (2,10)	9 (3,14)
**Trouble with coughing**	8 (5,11)	9 (6,11)	6 (3,9)	12 (8,16)	12 (7,16)	18 (12,23)	11 (5,16)	19 (9,28)
**Trouble talking**	2 (0,3)	2(1,4)	2 (0,4)	2 (1,4)	3 (0,6)	8 (3,12)	2 (0,4)	9 (2,15)

All scores range from 0-100. Higher scores on global health status and on functional scales indicate a positive effect, while higher scores on symptom scales indicate worse symptoms. A difference of 10 denotes clinical significance

Supplemental Table 2: Mean differences [MD: Arm B – Arm A] in health-related quality of life score between the treatment groups after neoadjuvant therapy and at 1 and 3 years of follow up. (only p values < 0·05 shown)

	Pre Surgery (n = 332)		1 Year Post Surgery (n = 191)		3 Year Post Surgery (n = 89)	
	MD (CI)	P-value	MD (CI)	P-value	MD (CI)	P-value
Global health status/QoL	-8 (-13,-4)	<0·001	3 (-2,8)	··	0 (-6,6)	··
**QLQ-C30 Functional Scales**						
Physical functioning	-6 (-8,-3)	<0·001	-1 (-5,3)	··	0 (-5,5)	··
Role functioning	-7 (-13,-2)	0·009	0 (-6,7)	··	-3 (-12,5)	··
Emotional functioning	-5 (-9,-1)	0·018	-5 (-9,0)	0·045	0 (-6,5)	··
Cognitive functioning	-1 (-4,3)	··	2 (-2,6)	··	-1 (-6,4)	··
Social functioning	-5 (-11,0)	0·039	5 (-2,11)	··	2 (-5,10)	
**QLQ-C30 Symptom Scales**						
Fatigue	10 (6,14)	0·005	-3 (-9,2)	··	-2 (-9,5)	··
Nausea and vomiting	1 (-2,5)	··	2 (-2,7)	··	1 (-4,7)	··
Pain	7 (3,11)	0·001	7 (2,13)	0·007	5 (-2,11)	··
Dyspnoea	4 (0,7)	0·046	3 (-2,8)	··	0 (-7,6)	··
Insomnia	-3(-9,2)	··	-2 (-8,5)	··	-4 (-12,4)	··
Appetite loss	8(2,14)	0·011	3 (-4,10)	··	0 (-9,9)	··
Constipation	5 (0,10)	··	4 (-2,9)	··	2 (-4,9)	··
Diarrhoea	2 (-1,6)	··	-5 (-11,0)	0·045	-8 (-15,-2)	0·016
Financial difficulties	3 (-2,8)	··	0 (-6,6)	··	-4 (-11,3)	··
**QLQ-OES 18 Symptom Scales**						
Eating	2 (-3,7)	··	3 (-3,9)	··	6 (-1,13)	··
Reflux	-1 (-6,3)	··	-2 (-7,3)	··	0 (-7,7)	··
Pain	3 (-1,7)	··	4 (-1,8)	··	1 (-5,6)	··
Trouble swallowing saliva	-3 (-9,2)	··	-3 (-8,3)	··	2 (-5,9)	
Choked when swallowing	-2 (-7,2)	··	-4 (-9,0)	··	4 (-2,9)	··
Dry mouth	1 (-5,7)	··	-5 (-12,3)	··	-2 (-10,7)	··
Trouble with taste	3 (-3,8)	··	-3 (-10,4)	··	-2 (-10,7)	··
Trouble with coughing	5 (1,10)	0·017	9 (3,15)	0·003	10 (2,18)	0·009
Trouble talking	0 (-2,2)	··	2 (-1,6)	··	5 (0,9)	··
Dysphagia	0 (-5,5)	··	0 (-6,5)	··	2 (-9,5)	··

Values in parentheses are 95% confidence intervals. Longitudinal mixed effects model utilized. A negative mean difference (MD) denotes better functional scales, and worse symptoms in Arm A (MAGIC/FLOT), and a positive score indicates better function and worse symptoms in Arm B (CROSS). An MD of 10 or more defines "clinical significance".
